# Program of Clinics Arranged for the Georgia Surgeons’ Club by the Surgeons of New Orleans, La., February 27-28, 1914

**Published:** 1914-01

**Authors:** 


### PROGRAM OF CLINICS ARRANGED FOR THE GEORGIA SURGEONS' CLUB BY THE SURGEONS OF NEW ORLEANS, LA., FEBRUARY 27, 28, 1914.

### Friday, February 27, 1914.

Presbyterian Hospital, 9:30 a. m.—Surgical Operations by Dr. J. M. Bachelor, Dr. W. T. Richards. Gynecological Operations by Dr. H. S. Cocram.

Touro Infirmary, 9:30 a. m.—Surgical Operations by Dr. E. D. Martin, Dr. Rudolph Matas, Dr. F. W. Parham. Gynecological Operations by Dr. W. Kohlmann, Dr. C. Jeff Miller.

Richardson Memorial, Tulane Campus 3 to 5 p. m.—Dr. C. W. Duval and Dr. M. Couret, Surgical Pathology. Dr. Gustav Mann, Surgical Physiology. Dr. Edmond Souchon, Anatomic Specimens.

Miles' Amphitheatre, Charity Hospital, 8 p. m.—Symposium 1. Arthritis—Dr. Homer Dupuy, ear, nose and throat; Dr. J. B. Elliot, Jr., medical; Dr. H. B. Gessner, surgical; Dr. Amedee Granger, X-ray; Dr. Jos. Hume, genito-urinary; Dr. Paul McIlhenny, orthopedic.

Symposium 2. Ascites—Dr. George S. Bel, Dr. J. T. Halsey and Dr. I. I. Lemann, medical; Dr. Rudolph Matas, surgical; Dr. C. J. Miller, gynecological.

Symposium 3. Spleno-Megaly—Dr. J. A. Lanford, pathology; Dr. F. W. Parham, surgery; Dr. Jos. D. Weis, medicine.

SATURDAY, FEBRUARY 28, 1914.

Charity Hospital, 9:00-11:00 a. m.—Surgical Operations by Drs. C. W. Allen, S. P. Deleaup, J. A. Danna, F. Larue, R. Matas, U. Maes, F. W. Parham, J. Smyth, M. Souchon, S. W. Stafford. Gynecological by Drs. S. M. D. Clark, E. S. Lewis, P. Michinard, C. J. Miller. Genito-urinary, Dr. A. Nelken.

Ward Clinic, 9:00-11:30.—Drs. Hume and Logan, Syphilis. Orthopedic Clinics by Dr. E. D. Fenner, Dr. E. S. Hatch, Dr. J. F. Oechsner.

11:30-1:30.—Presentation of interesting cases with ten minutes talk by the visiting Hospital staff.

Drs. C. W. Allen, G. S. Bel, C. G. Cole, O. Joachim, C. J. Landfried, R. Lyons, R. Matas, E. W. Mahler, H. Menage, J. Oechsner, F. W. Parham, W. M. Perkins, and others.

Hutchinson Memorial, 2:30 p. m.—Demonstrations in the Laboratory of Experimental Surgery, Laboratory of Operative Surgery, and Laboratory of Tropical Medicine (Dr. C. Wellman).

